# Improving Medication Classification in GLP‐1RA Research: Feasibility of a Photo‐Review Protocol

**DOI:** 10.1002/osp4.70200

**Published:** 2026-09-22

**Authors:** Jacqueline M. Katz, Ashley N. Gearhardt, Dina H. Griauzde

**Affiliations:** ^1^ Department of Psychology University of Michigan Ann Arbor Michigan USA; ^2^ VA Ann Arbor Healthcare System Ann Arbor Michigan USA; ^3^ Department of Internal Medicine University of Michigan Medical School Ann Arbor Michigan USA; ^4^ University of Michigan Institute for Healthcare Policy and Innovation Ann Arbor Michigan USA

**Keywords:** GLP‐1RA, medication verification, methods, online research protocol

## Abstract

**Objective:**

Glucagon‐like peptide‐1 receptor agonists (GLP‐1RAs) are dominating the type 2 diabetes and obesity intervention landscape, yet their real‐world use is increasingly difficult to characterize amid widespread availability of non–FDA‐approved, compounded products obtained outside traditional healthcare systems. These users may not be captured by the clinical and claims databases commonly used in real‐world GLP‐1RA research. Online surveys offer a scalable alternative for reaching this population; however, existing survey studies depend on self‐reported medication use, the reliability of which has not been well characterized.

**Methods:**

An online study was conducted among U.S. adults recruited via Prolific who reported ≥ 3 months use of once‐weekly injectable semaglutide or tirzepatide. Participants completed a screening survey requiring a photo of the prescribed GLP‐1RA, followed by a main survey for eligible participants assessing self‐reported GLP‐1RA type and sociodemographic and health characteristics. Two blinded, independent coders classified photos as one of four FDA‐approved branded formulations or a compounded product; discrepancies were resolved by the second author. Agreement was assessed using Cohen's kappa.

**Results:**

Of the 419 screening survey participants, 322 participants comprised the analytic sample; 22 of the total exclusions were based on medication photo review. Self‐reported and photo‐coded medication types showed substantial agreement (82.61%; Cohen's *κ* = 0.78, 95% CI [0.73, 0.83]). Photo review classified 83 participants (25.78%) as using compounded GLP‐1RAs; among these, 54 (65.06%) self‐reported using one of the four branded GLP‐1RAs and 29 (34.94%) selected a “none of the above” response option, operationalized as compounded use.

**Conclusions:**

This study demonstrated that photo review may be a feasible method for improving medication classification in research and offered recommendations for future studies. Additional work is needed to evaluate this protocol in larger and more diverse samples and to refine formulation‐specific self‐report items.

## 1 Introduction

The U.S. Food and Drug Administration's (FDA's) approvals of semaglutide and tirzepatide for weight management—collectively referred to as glucagon‐like peptide‐1 receptor agonists (GLP‐1RAs)—led to a sharp increase in demand for these therapies. During the resulting shortages, GLP‐1RA access expanded beyond traditional healthcare systems, including through telehealth platforms and esthetic spas offering non‐FDA‐approved compounded GLP‐1RA formulations. Although the shortages have since resolved, high costs and other barriers to access continue to contribute to medication sourcing through these alternative channels [1, 2, 3]. As a result, conventional approaches to investigating medication use—including electronic health record‐ and claims‐based studies—may not accurately reflect real‐world use and fail to capture a substantial portion of GLP‐1RA users. This limitation is particularly important for compounded products, which may differ from their branded, FDA‐approved counterparts in dose, composition, safety, and effects [2, 4]. Therefore, feasible, reliable, and scalable methods are needed to better characterize GLP‐1RAs in research.

Despite the clinical uptake and public interest in compounded GLP‐1RAs, little is known about how to evaluate their use in real‐world settings. Limited prior work has used unstructured data, such as clinic notes, or retrospective evaluation of patients with known compounded prescriptions [5, 6]. Online survey research offers one approach for reaching and efficiently recruiting GLP‐1RA users across care settings. However, existing online studies have relied on self‐reported GLP‐1RA use, the reliability of which remains insufficiently characterized [7, 8, 9, 10, 11, 12, 13, 14].

The present study evaluated the feasibility of incorporating medication photo review into online GLP‐1RA research. The present study examined whether an online protocol requiring participants to upload deidentified medication photos could be implemented successfully, whether uploaded images could be reviewed and coded using a structured workflow, and whether self‐reported medication type aligned with photo‐based classification. Another aim was to develop practical recommendations for future online GLP‐1RA studies. This was the first study to describe a replicable photo‐review workflow for online GLP‐1RA studies and characterize patterns of agreement and discrepancy between participant self‐report and adjudicated photo‐coded medication type.

## 2 Methods

### 2.1 Study Design

A cross‐sectional online study of GLP‐1RA use recruited participants between October 2025 and January 2026 through Prolific, an online research subject pool shown to yield high‐quality data [15]. Participants first completed a brief screening survey in Qualtrics to assess GLP‐1RA use based on self‐report and photo documentation of the prescribed GLP‐1RA. Eligible participants then completed a second, main survey in Qualtrics assessing sociodemographic and basic anthropometric characteristics as well as medication‐related information, including GLP‐1RA formulation, prescriber, and primary payer type.

### 2.2 Participants and Prolific Prescreening

Eligibility criteria were designed to recruit U.S.‐based GLP‐1RA users and ensure high‐quality data. Prolific prescreening filters restricted screening survey access to individuals who: (1) reside in the United States; (2) have a Prolific approval rate ≥ 99%; (3) self‐report current Ozempic or other GLP‐1RA use; and (4) have completed ≥ 50 previous submissions in Prolific.

A brief screening survey was made available on Prolific to participants who met the prescreening criteria. To minimize ineligible screening submissions, the Prolific study description and the first page of the screening survey instructed participants to proceed only if they were able to take and upload a photo documenting their current GLP‐1RA.

### 2.3 Screening Survey and Eligibility

The screening survey included three self‐report multiple‐choice items (see Supporting Information S1: Appendix Methods) and required participants to upload a photo of their current GLP‐1RA. The first two items assessed current use of an injectable GLP‐1RA containing semaglutide or tirzepatide, such as Ozempic, Wegovy, Mounjaro, or Zepbound, and once‐weekly administration of the current medication. Both items had yes/no response options. Participants then reported how long they had been using their current once‐weekly GLP‐1RA, with response options ranging from less than 3 months to more than 2 years. Participants were eligible for the main survey if they reported current use of an injectable medication containing semaglutide or tirzepatide, once‐weekly administration, and ≥ 3 months use.

**FIGURE 1:**
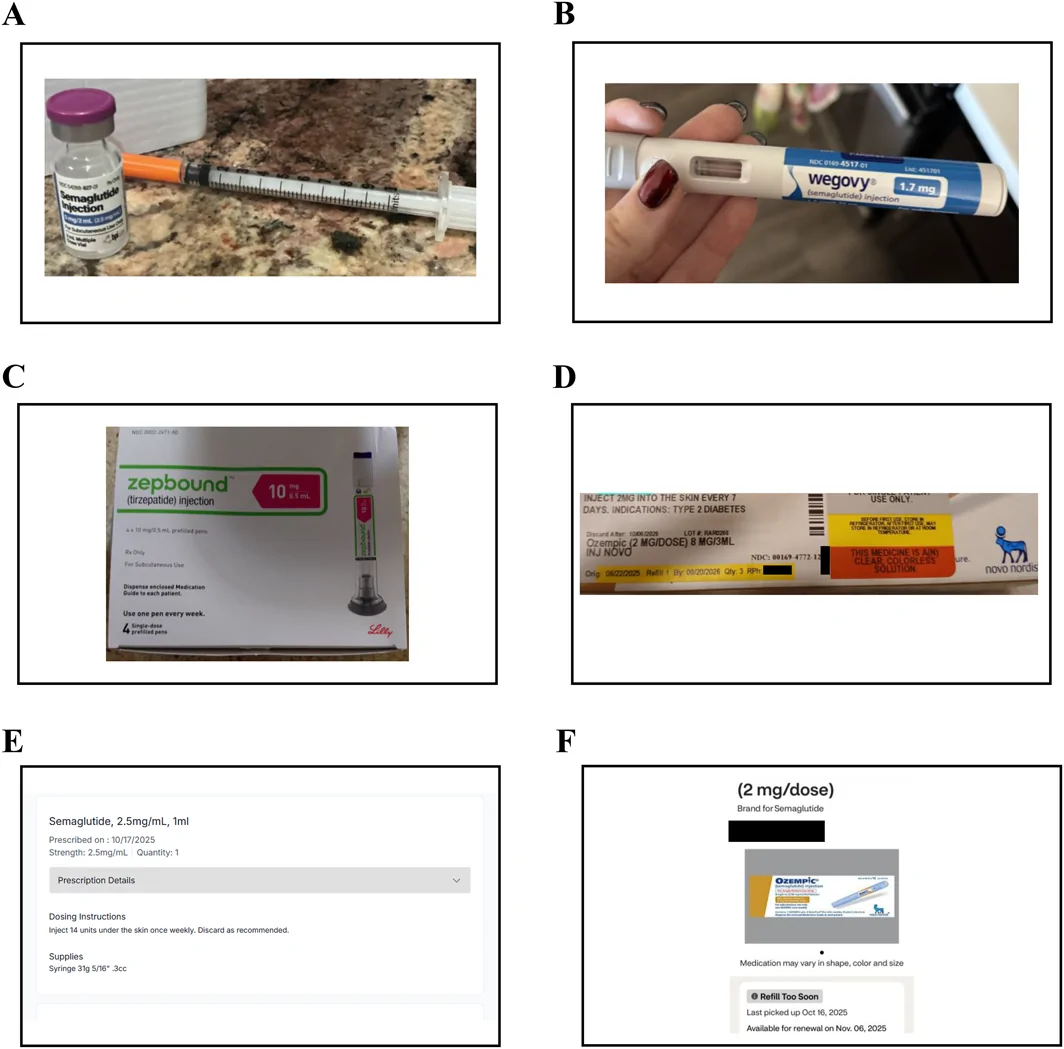
Examples of accepted deidentified medication documentation. Panels depict actual examples of documentation submitted by participants that were accepted for use in eligibility assessment and medication classification, including medication photos (A and B), medication label (C), pharmacy label (D), prescription documentation (E), and medication documentation generated through an application (F). Personal identifying information was removed before storage, analysis, and inclusion in the figure.

Eligibility assessment included photo documentation of the current GLP‐1RA. Accepted documentation included photos of the medication itself (i.e., injector pen, vial, and/or syringe). When the medication was not directly pictured, documentation was also accepted in the form of a medication label, pharmacy label, prescription document, and/or medication documentation generated through pharmacy, telehealth platform, or weight management applications. See Figure 1 for example submissions. Participants were excluded if the uploaded photo appeared to be a stock image, was inaccessible, depicted an unidentifiable medication, or showed a medication other than an injectable medication containing semaglutide or tirzepatide.

The first author reviewed uploaded photos and screening survey responses. Participants who met eligibility criteria were invited to complete the main survey.

### 2.4 Main Survey Instrument and Measures

Participants completed a series of self‐report questionnaires assessing sociodemographic and anthropometric characteristics as well as medication‐related information, including GLP‐1RA type, prescriber category, and primary payer type. All items reported herein were forced choice, with the exception of age, pre‐treatment weight, and current weight.

Subjective social status used to describe the sample was assessed using the MacArthur Scale of Subjective Social Status. Scores range from 1 to 10, with higher scores representing higher perceived social status [16].

Anthropometric items included current height, current weight, and weight immediately before initiating the current GLP‐1RA, which were used to describe the sample. Body mass index (BMI) was calculated using the standard formula for imperial units: weight in pounds divided by height in inches squared, multiplied by 703. Pre‐treatment BMI was calculated using the reported weight immediately before GLP‐1RA initiation and current reported height. Current BMI was calculated using current reported weight and current reported height.

#### 2.4.1 Self‐Reported Medication Type

In the main survey, participants self‐reported current GLP‐1RA medication type using the item: “What type of GLP‐1 medication do you currently use?” Response options were “Ozempic (semaglutide),” “Wegovy (semaglutide),” “Mounjaro (tirzepatide),” “Zepbound (tirzepatide),” “I am not sure,” and “None of the above (please specify),” with a free‐text field for participants who selected “None of the above (please specify).” The selected response to this item was used to define the participants' self‐reported GLP‐1RA type. No participant selected “I am not sure.”

#### 2.4.2 Data Quality Procedures

Data quality procedures included Qualtrics bot detection, attention checks, duplicate prevention, and review of response patterns. Qualtrics bot detection uses Google reCAPTCHA technology to generate a score from 0.0 to 1.0, with lower scores indicating greater likelihood of automated activity. Consistent with Google's recommended default threshold, responses with reCAPTCHA scores below 0.50 were flagged for further review, including review of attention checks, completion times, and responses to multiple‐choice and write‐in items [17]. Three attention checks were embedded throughout the main survey: (1) a multiple‐choice question asking participants to identify which item would *not* be considered an ultraprocessed food, administered after reviewing distinctions between ultraprocessed and minimally processed foods; (2) a slider item instructing participants to move a bar to 100; and (3) a multiple‐choice item instructing participants to select a prespecified response option. Prolific ID, study ID, and session ID were recorded to prevent duplicate responses, and platform settings were configured to allow each participant to complete the screening survey and main survey only once. Submissions falling significantly below the estimated completion time were flagged for automatic rejection.

### 2.5 Medication Photo Review Protocol

#### 2.5.1 Privacy Procedures

To protect participant privacy with respect to the medication photo, participants were instructed to “Please take a picture of your current GLP‐1 medication. Upload a single picture in the section below, showing the label but hiding any personal identifying information (e.g., name, date of birth).” The first author reviewed the photos for any identifiable information. The first author then edited photos containing identifiable information (i.e., face, name, address, prescriber information, or pharmacy information) to remove any identifiable information, deleted the unredacted originals from Qualtrics, and saved the redacted copies in a secure folder accessible only to authorized study personnel.

#### 2.5.2 Medication Photo Coding

For all main survey participants, medication documentation uploaded during the screening survey was subsequently coded by medication type. Photos were independently reviewed by two trained research staff coders who had not participated in eligibility or privacy review and were blinded to participants' self‐reported medication type. Discrepancies between the two primary coders were reviewed by the second author, an independent adjudicator who had not previously reviewed the photos and was blinded to participant self‐report and the primary coders' classifications. Medication type was coded into one of five mutually exclusive categories: Ozempic, Wegovy, Mounjaro, Zepbound, or compounded medication containing semaglutide or tirzepatide. A medication was coded as compounded semaglutide or tirzepatide when the uploaded medication photo showed one of these active pharmaceutical ingredients and did not include a brand name. A medication was coded as one of the FDA‐approved branded formulations when the documentation identified Ozempic, Wegovy, Mounjaro, or Zepbound on the medication itself or on associated labeling or records.

### 2.6 Statistical Analysis

Analyses were conducted in IBM SPSS Statistics (version 31.0). All tests were two‐sided with *α* = 0.05. Descriptive statistics were conducted to characterize the sample and study variables.

Agreement between participant self‐reported GLP‐1RA type on the main survey and adjudicated photo‐based classification was quantified to evaluate the performance of the medication photo‐review protocol. For this analysis, the four branded response options were retained as distinct self‐reported medication categories and treated as concordant with photo‐based classifications of Ozempic, Wegovy, Mounjaro, or Zepbound. Because the analytic sample was restricted to participants who had already reported current use of an injectable semaglutide‐ or tirzepatide‐containing medication, responses of “None of the above (please specify)” were operationalized as self‐reported use of a compounded semaglutide‐ or tirzepatide‐containing product. Accordingly, “None of the above (please specify)” was treated as concordant with the photo‐based classification of compounded GLP‐1RAs.

A secondary analysis collapsed medication types into branded and compounded groups. Treating adjudicated photo‐based medication classification as the reference and compounded use as the positive condition, sensitivity was calculated as the proportion of photo‐coded compounded users classified as compounded by self‐report; specificity as the proportion of photo‐coded branded users classified as branded by self‐report; positive predictive value (PPV) as the proportion of self‐reported compounded users classified as compounded by photo review; and negative predictive value (NPV) as the proportion of self‐reported branded users classified as branded by photo review.

Agreement was summarized using percent agreement and Cohen's kappa (*κ*) with 95% confidence intervals; kappa was computed on the analytic sample (*N* = 322). Kappa values were interpreted using conventional benchmarks (e.g., 0.41–0.60 moderate, 0.61–0.80 substantial, 0.81–1.00 almost perfect).

### 2.7 Ethical Considerations

The research was deemed exempt by the University of Michigan Health Sciences and Behavioral Sciences Institutional Review Board (HUM00270291). Electronic informed consent was obtained from all participants for both the screening survey and the main survey. Participants provided informed consent acknowledging that study results could be published or presented but would not include information that could identify them to others. On average, compensation was $10.05/hour for the screening survey and $14.99/hour for the main survey.

## 3 Results

### 3.1 Sample Characteristics

Prolific prescreening filters identified 1209 participants who met the platform‐based prescreening criteria. Of these, Qualtrics recorded 432 screening survey entries. After removal of 6 duplicate and 7 incomplete entries, 419 non‐duplicate, completed screening survey responses remained, corresponding to 34.7% of the Prolific‐prescreened eligible pool.

**FIGURE 2:**
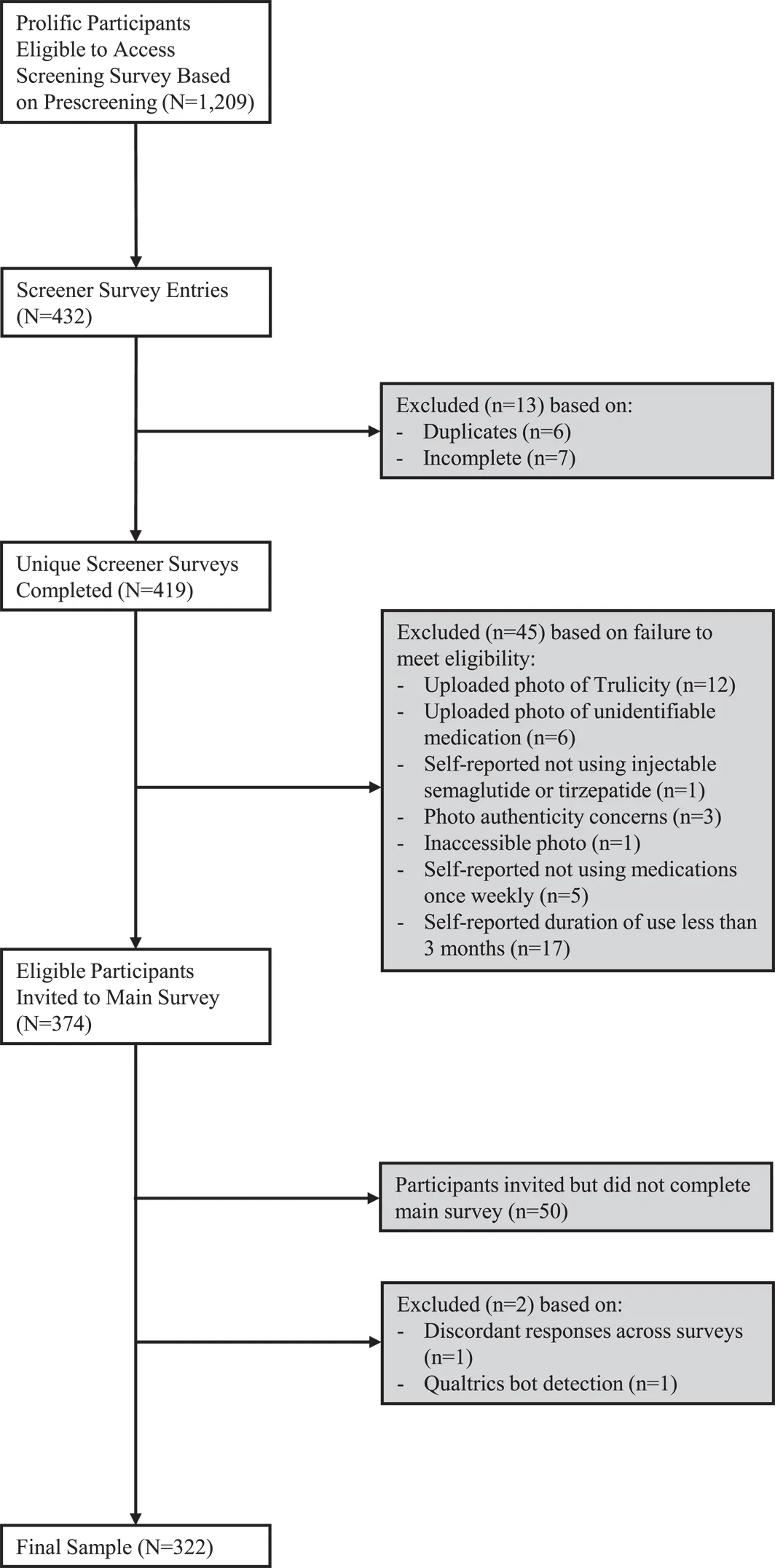
Participant flow through screening, eligibility review, and analytic sample Formation. Flow diagram showing the implementation of exclusion criteria and participants included across stages of the two‐survey study design.

Figure 2 depicts participant flow and the implementation of exclusion criteria across the two‐survey study. Of the 419 screening survey completers, 47 participants were excluded from the study for the following reasons: failing to meet the minimum duration criterion of ≥ 3 months of use (*n* = 17), uploading a photo of an ineligible medication (*n* = 12; all depicted Trulicity), failing to meet the once‐weekly dosing frequency criterion (*n* = 5), photo authenticity concerns (*n* = 3; images appeared to be stock photos), uploading an inaccessible medication photo (*n* = 1), uploading a photo of an unidentifiable medication (*n* = 6), denying current use of a semaglutide or tirzepatide injectable (*n* = 1), inconsistent self‐report responses across surveys (*n* = 1), and failing Qualtrics bot detection (*n* = 1). The participant excluded after bot‐detection/data‐quality review had multiple indicators of low‐quality data, including a reCAPTCHA score of 0.1, a completion time of exactly 30:00, an unlikely response pattern, and an incorrect response to the attention check assessing understanding of ultraprocessed versus minimally processed foods. All study completers, except for the one excluded after bot‐detection/data‐quality review, passed each attention check. No participants were excluded from exceptionally fast submissions. In total, 22 participants were excluded based on their medication photos. Fifty screening survey participants who were invited to participate in the main survey did not complete it.

The analytic sample included 322 participants who self‐reported once‐weekly injectable semaglutide‐ or tirzepatide‐containing medication use for at least 3 months and uploaded an acceptable photo documenting an eligible medication. Demographic and clinical characteristics are described in Table 1.

**TABLE 1:** Sample demographics and clinical characteristics.

	Mean	SD	Range
Age (in years)	45.06	12.14	22–76
Pre‐treatment BMI	40.11	9.34	21.03–76.26
Current BMI	32.87	9.05	15.21–69.50
Subjective social status	5.42	1.63	1–9

	***n***	%	
Race			
American Indian/Native American or Alaska Native	2	0.6	
Asian	9	2.8	
Black or African American	26	8.1	
White or Caucasian	260	80.7	
More than one race	19	5.9	
Other	6	1.9	
Ethnicity			
Hispanic, Latino, or Spanish origin	36	11.2	
Not Hispanic, Latino, or Spanish origin	286	88.8	
Educational attainment			
High school diploma or GED	20	6.2	
Some college, but no degree	57	17.7	
Associates or technical degree	43	13.4	
Bachelor's degree	126	39.1	
Graduate or professional degree (MA, MS, MBA, PhD, JD, MD, DDS, etc.)	76	23.6	
Sex			
Male	74	23.0	
Female	248	77.0	
GLP‐1RA type			
Ozempic	62	19.3	
Wegovy	43	13.4	
Mounjaro	68	21.1	
Zepbound	66	20.5	
Compounded semaglutide or tirzepatide	83	25.8	
GLP‐1RA treatment duration			
3–6 months	76	23.6	
7–9 months	53	16.5	
10–12 months	53	16.5	
13–18 months	65	20.2	
19–24 months	39	12.1	
> 24 months	36	11.2	
Primary payer type			
Private	221	68.6	
Medicare	48	14.9	
Medicaid	35	10.9	
Self‐pay	13	4.0	
Other	5	1.6	
Prescriber type			
Primary care provider	173	53.7	
Specialist	53	16.5	
Telehealth/online provider	73	22.7	
Clinician through manufacturer program	2	0.6	
Medical spa or esthetic center	11	3.4	
Other	10	3.1	

*Note: N* = 322. GLP‐1RA type (i.e., Ozempic, Wegovy, Mounjaro, Zepbound, and compounded semaglutide or tirzepatide) refers to adjudicated photo‐coded medication type. Other demographic and health‐related information is based on participant responses to survey items.

### 3.2 Photo Coding Feasibility

Uploaded medication photos generally provided sufficient information for coders to classify medication types. During screening, six participants uploaded photos that were accessible but did not provide enough information for photo‐based medication classification; these participants were excluded before the analytic sample was finalized. Within the analytic sample (*N* = 322), intercoder agreement before adjudication was almost perfect (Cohen's *κ* = 0.98, 95% CI [0.96, 1.00]), with five coding discrepancies resolved by adjudication. The discrepant cases included one photo of a Mounjaro injector pen with a label in a foreign language, one photo of a compounded semaglutide product, and three photos of branded products (Wegovy, *n* = 1; Mounjaro, *n* = 2).

### 3.3 Agreement Between Self‐Reported and Photo‐Coded GLP‐1RA Types

Table 2 shows the agreement between self‐reported and adjudicated photo‐coded medication types. Self‐reported medication type showed substantial agreement with photo‐coded medication type in the full sample (Cohen's *κ* = 0.78, 95% CI [0.73, 0.83]). Overall, 266 of the 322 participants were classified concordantly by self‐report and photo coding, yielding 82.61% agreement between the two methods. 56 participants had discrepant self‐reported and photo‐coded medication classifications.

**TABLE 2:** Agreement between self‐reported and photo‐coded GLP‐1RA type.

		Adjudicated photo‐coded GLP‐1RA type					
		Ozempic	Wegovy	Mounjaro	Zepbound	Compounded GLP‐1RA	Total
Self‐reported GLP‐1RA type	Ozempic	**62**	0	0	0	19	81
	Wegovy	0	**43**	1	0	5	49
	Mounjaro	0	0	**67**	1	15	83
	Zepbound	0	0	0	**65**	15	80
	None of the above	0	0	0	0	**29**	29
Total		62	43	68	66	83	322

*Note:* Values are *n*. Bolded, green‐shaded diagonal cells represent agreement between self‐report and adjudicated photo‐based classification on GLP‐1RA type. “None of the above” refers to participants who selected “None of the above (please specify)” in the main survey; these responses were treated as self‐reported compounded semaglutide or tirzepatide use.

When medication type was collapsed into branded and compounded groups, self‐reported medication type showed moderate agreement with photo‐coded medication type (Cohen's *κ* = 0.44, 95% CI [0.33, 0.56]). Here, self‐report sensitivity was 34.94% (29/83), specificity was 100% (239/239), PPV was 100% (29/29), and NPV was 81.57% (239/293; see Supporting Information S1: Appendix Table A).

Discrepancies were most common among participants whose uploaded photos were coded as compounded semaglutide‐ or tirzepatide‐containing products. Based on adjudicated photo coding, 83 participants (25.78%) were classified as using a compounded semaglutide‐ or tirzepatide‐containing product. Within this photo‐coded compounded group, 29 participants (34.94% of compounded users; 9.01% of the total analytic sample) self‐reported “None of the above (please specify)” and provided written responses consistent with the use of compounded semaglutide‐ or tirzepatide‐containing products; these responses were treated as concordant with the photo‐based classification of compounded GLP‐1RA use. Themes of written responses are provided in Supporting Information S1: Appendix Table B.

The remaining 54 participants in the photo‐coded compounded group (65.06%) self‐reported using branded medications, resulting in discordance between self‐reported and photo‐coded medication types. These participants self‐reported the use of Ozempic (*n* = 19), Wegovy (*n* = 5), Mounjaro (*n* = 15), or Zepbound (*n* = 15).

The only two discrepancies not involving compounded products were brand‐to‐brand discrepancies: one participant was photo‐coded as using Mounjaro but self‐reported Wegovy use, and one participant was photo‐coded as using Zepbound but self‐reported Mounjaro use.

## 4 Discussion

This study demonstrated the feasibility of incorporating medication photo review into online GLP‐1RA research to strengthen characterization of medication exposure. An online recruitment platform and survey workflow were used to recruit participants reporting GLP‐1RA use, collect deidentified medication photos during screening, implement privacy protections for submitted medication photos, and classify medication type using a blinded dual‐coder review process with adjudication. These findings suggest that deidentified medication photo review may be a feasible and scalable strategy for improving medication classification in online studies of GLP‐1RA use.

Participants uploaded photos that generally provided sufficient information to determine medication type, and independent coders demonstrated almost perfect agreement before adjudication. These findings are important because online GLP‐1RA research is likely to become increasingly common as use continues to expand across both traditional clinical settings and nontraditional care pathways. A structured photo‐review protocol may support eligibility determination and strengthen medication characterization.

This study also demonstrated why a photo review may be valuable in online GLP‐1RA research. Although self‐report showed substantial concordance with photo‐coded medication classification, discrepancies were common among participants using compounded GLP‐1RAs. Among participants whose photos were coded as compounded semaglutide‐ or tirzepatide‐containing products, 65.06% self‐reported the use of a branded medication. This pattern suggests that self‐report alone may not reliably distinguish branded from compounded GLP‐1RA users and is consistent with prior evidence that the validity of self‐reported medication use is generally high but varies across medication classes and contexts [18]. It also aligns with a recent GLP‐1RA study in which electronic health record data identified a higher proportion of compounded use than participant self‐report [14]. The present study extended this work to a larger online sample that may include users not captured through electronic health records and demonstrated the feasibility of a photo‐review protocol to strengthen GLP‐1RA classification.

These findings have important implications for studies in which GLP‐1RA formulation or source is clinically meaningful. To date, published online studies and polls have used self‐report to define GLP‐1RA user groups, many providing limited detail on how use status was determined, and few assessing specific medication type through self‐report (e.g., semaglutide, tirzepatide) [7, 8, 9, 10, 11, 12, 13, 14]. Review procedures are increasingly important where medication formulation is clinically meaningful, particularly in the United States, where compounded semaglutide‐ or tirzepatide‐containing products remain widely accessible outside traditional health systems. Notably, photo‐coded compounded products represented more than one quarter of the present sample despite data collection occurring after semaglutide and tirzepatide shortages were resolved, emphasizing the continued importance of medication review beyond the shortage period. In this context, reliance on self‐report alone may not reliably classify medication type, potentially biasing inferences about medication effects and user experiences.

The approach presented herein adapted established medication‐verification practices to online GLP‐1RA research. This protocol conceptually borrowed from “brown bag” medication reviews in clinical care and from research in the field of reproductive endocrinology (e.g., participants bring oral contraceptive pill packs into study visits to verify specific hormonal formulations) [19, 20]. The online photo‐review workflow preserved the core logic of medication verification while reducing participant burden and enabling recruitment of GLP‐1RA users across diverse geographic and care settings. As GLP‐1RA use grows outside traditional healthcare settings, methodology must keep pace with evolving patterns of use across clinical contexts. Deidentified medication photo reviews offer one practical way to strengthen online research while preserving participant privacy.

The present findings offer a number of practical recommendations for future online GLP‐1RA studies. When medication type or formulation is central to the research question, research teams should consider using medication photo review. Additionally, when medication use determines eligibility, a manual photo review should be conducted during screening as part of a two‐stage study design. Participants should be informed in advance that they will be required to upload a photo of their current medication to limit incomplete surveys, time‐outs, and participant attrition. Photo‐upload instructions should: (1) direct participants to keep the medication name, brand name, or active ingredient visible; (2) list acceptable forms of medication documentation; and (3) direct participants to remove personal identifiers before upload. Future studies may also instruct participants to photograph the medication with the survey visible in the background to reduce concerns about photo authenticity. To further protect participant privacy, study staff should review uploaded photos for identifiers, save only redacted copies, and delete originals containing identifiable information. In studies with self‐reported medication type, items should include explicit response options for compounded products and undergo pilot testing to assess how participants understand formulation terminology. Finally, studies should use independent, blinded dual coding with adjudication when feasible and report inter‐rater reliability, photo‐review outcomes, and photo‐based exclusions.

Despite its strengths, this study had several limitations. First, stringent prescreening filters in Prolific identified only 1209 eligible participants of the platform's > 300,000 verified users. Participants with high approval rates and prior Prolific experience may have been more attentive to study instructions and more able or willing to provide acceptable documentation. As such, results may not generalize to all online samples, and the extent of discordance observed here was likely conservative. Additional work is needed to evaluate this protocol in larger and more diverse samples and to determine whether photo review affects participation rates, sample composition, or participant trust.

Second, the survey did not include an explicit response option for participants to self‐report the use of a compounded product containing semaglutide or tirzepatide. The medication‐type item listed only FDA‐approved branded products containing these active ingredients—Ozempic, Wegovy, Mounjaro, and Zepbound—along with “I am not sure” and “None of the above (please specify).” Because participants had already reported the current use of an injectable medication containing semaglutide or tirzepatide, the selection of “None of the above (please specify)” was treated as concordant with the photo‐based classification of compounded medications. The absence of an explicit response option for compounded formulations may have contributed to the observed discordance among photo‐coded compounded users, thereby limiting conclusions as to whether this pattern reflects self‐report unreliability or limitations of the instrument itself. Future studies should use cognitive interviewing, randomized survey wording experiments, or other instrument‐development methods to test how participants understand and respond to different phrasing, including terms such as “compounded” and “non‐branded.” Preliminary results from this study suggested that participants may understand response options that include “compounded” and “generic” language (see Supporting Information S1: Appendix Table B).

Third, photo‐based classification was not externally validated against dispensing records or chemical assays and therefore should not be considered a ground‐truth measure of medication exposure. Uploaded medication photos were coded as branded products (i.e., Ozempic, Wegovy, Mounjaro, or Zepbound) when the medication itself or associated labeling and records displayed the corresponding brand name. Thus, photo review allowed classification based on the information visible in the submitted documentation but cannot confirm the authenticity or contents of the medication.

## 5 Conclusion

This study demonstrated the feasibility of a medication photo‐review workflow to improve the classification of real‐world GLP‐1RA use in online research. The protocol supported eligibility assessment, structured photo coding, strong intercoder agreement, and identification of discrepancies between self‐reported and photo‐coded medication types, though future work should evaluate this protocol in larger and more diverse samples and refine self‐report items designed to distinguish medication formulations. In this study, self‐reporting did not reliably distinguish compounded semaglutide‐ and tirzepatide‐containing products from FDA‐approved branded formulations. As GLP‐1RA use continues to expand across traditional and nontraditional care settings, structured review procedures may improve medication exposure classification and strengthen the validity of inferences drawn from online studies.

## Author Contributions

**Jacqueline M. Katz:** study design, data collection, statistical analysis, writing original draft, and reviewing and editing. **Ashley N. Gearhardt:** supervision, study design, and reviewing and editing. **Dina H. Griauzde:** supervision, study design, and reviewing and editing. All authors approved the final manuscript for publication.

## Funding

This study was supported by the University of Michigan Eisenberg Family Depression Center and University of Michigan Department of Psychology. Dr. Gearhardt's time was also supported by a grant from the National Institute on Drug Abuse (R01DA054750; principal investigator: A. N. Gearhardt), and Jacqueline Katz's time was supported by a Eunice Kennedy Shriver National Institute of Child Health and Human Development Developmental Psychology Training Grant (5T32HD007109; principal investigator: C.S. Monk).

## Conflicts of Interest

Dr. Griauzde reports research grant support from NIH/NIDDK and Eli Lilly; consulting and administrative support from the Michigan Collaborative for Type 2 Diabetes and the Caswell Diabetes Institute; and honoraria from Blue Cross Blue Shield of Michigan, the American Diabetes Association, the Michigan Bariatric Society, and Medscape/WebMD. Dr. Gearhardt has received speaking honoraria from academic organizations and health related nonprofits, consulting fees from health‐related nonprofits and a law firm and receives royalties from Oxford University Press. All other authors declare no competing interests.

## Supporting information


Supporting Information S1

## Data Availability

The data that support the findings of this study are available from the corresponding author upon reasonable request.
