# Supplementary material for: Improving Medication Classification in GLP‐1RA Research: Feasibility of a Photo‐Review Protocol
Source: Obes Sci Pract. 2026 Sep 22;12(5):e70200. doi: 10.1002/osp4.70200 (PMC13595039; doi:10.1002/osp4.70200)
Supplement: Supplementary file 1 [file OSP4-12-e70200-s001.docx]

eMethods 1. Screener Multiple-Choice Instrument Items

Are you **currently** using an injectable GLP-1 medication containing semaglutide or tirzepatide (e.g., Ozempic, Wegovy, Mounjaro, Zepbound)?

- Yes
- No

Are you taking the **current** GLP-1 medication once per week?

- Yes
- No

How long have you been using the **current** GLP-1 medication once per week?

- Less than 3 months
- 3-6 months
- 7-9 months
- 10-12 months
- 13-18 months
- 19-24 months
- More than 2 years
- I do not currently use a GLP-1 medication once per week

Table A. Agreement Between Self-Reported and Photo-Coded GLP-1RA Type: Branded vs Compounded Products

|  | | Adjudicated Photo-Coded GLP-1RA Type | | |
| --- | --- | --- | --- | --- |
|  |  | Branded | Compounded | Total |
| Self-Reported GLP-1RA Type | Branded | **239** | 54 | 293 |
|  | Compounded (“None of the Above”) | 0 | **29** | 29 |
| Total | | 239 | 83 | 322 |

Note. Values are n. Bolded, green-shaded diagonal cells represent agreement between self-report and adjudicated photo-based classification on GLP-1RA type. Branded products included Ozempic, Wegovy, Mounjaro, and Zepbound. “None of the Above” refers to participants who selected “None of the above (please specify)” in the main survey; these responses were treated as self-reported compounded semaglutide or tirzepatide use.

Table B. Free-Text Descriptors of GLP-1RA Type Among Participants Selecting “None of the Above”

| Descriptor appearing in free text | N (%) |
| --- | --- |
| “compounded,” “compound,” or “compounding” | 20 (69.0%) |
| “generic” | 7 (24.1%) |
| “unbranded,” “not name branded,” “not have a brand name,” or “no name brand” | 4 (13.8%) |
| Named specific telehealth platform and/or compounding pharmacy | 6 (20.7%) |
| “tirzepatide” or “semaglutide” | 27 (93.1%) |

Note. Descriptors reflect the language used to characterize GLP-1RA medication among participants who selected "None of the above (please specify)" for medication type (N=29). Values indicate the number and percentage of participants whose responses included each descriptor. Descriptor categories are not mutually exclusive.
